# Circulating Microparticles Alter Formation, Structure, and Properties of Fibrin Clots

**DOI:** 10.1038/srep17611

**Published:** 2015-12-04

**Authors:** Laily D. Zubairova, Roza M. Nabiullina, Chandrasekaran Nagaswami, Yuriy F. Zuev, Ilshat G. Mustafin, Rustem I. Litvinov, John W. Weisel

**Affiliations:** 1 Department of General Pathology, Kazan State Medical University, Kazan 420012, Russian Federation; 2 Department of Cell and Developmental Biology, University of Pennsylvania School of Medicine, Philadelphia, Pennsylvania 19104, USA; 3 Institute of Biochemistry and Biophysics, Russian Academy of Sciences, Kazan 420111, Russian Federation; 4 Institute of Fundamental Medicine and Biology, Kazan Federal University, Kazan 420012, Russian Federation

## Abstract

Despite the importance of circulating microparticles in haemostasis and thrombosis, there is limited evidence for potential causative effects of naturally produced cell-derived microparticles on fibrin clot formation and its properties. We studied the significance of blood microparticles for fibrin formation, structure, and susceptibility to fibrinolysis by removing them from platelet-free plasma using filtration. Clots made in platelet-free and microparticle-depleted plasma samples from the same healthy donors were analyzed in parallel. Microparticles accelerate fibrin polymerisation and support formation of more compact clots that resist internal and external fibrinolysis. These variations correlate with faster thrombin generation, suggesting thrombin-mediated kinetic effects of microparticles on fibrin formation, structure, and properties. In addition, clots formed in the presence of microparticles, unlike clots from the microparticle-depleted plasma, contain 0.1–0.5-μm size granular and CD61-positive material on fibres, suggesting that platelet-derived microparticles attach to fibrin. Therefore, the blood of healthy individuals contains functional microparticles at the levels that have a procoagulant potential. They affect the structure and stability of fibrin clots indirectly through acceleration of thrombin generation and through direct physical incorporation into the fibrin network. Both mechanisms underlie a potential role of microparticles in haemostasis and thrombosis as modulators of fibrin formation, structure, and resistance to fibrinolysis.

Circulating microparticles (MPs) are 0.1–1-μm-large phospholipid vesicles^1^ released from blood and vascular cells upon activation and apoptosis. The mechanism of MP formation by budding of the outer cell membranes provides them with procoagulant activity, mainly due to phosphatidylserine exposure and tissue factor expression^2^,^3^. Tissue factor-bearing MPs are important for thrombin generation and blood clotting *in vitro*^4^ as well as for thrombus formation *in vivo*^5^,^6^,^7^,^8^,^9^,^10^.

MPs are present in the blood under physiological conditions, but the level of circulating MPs is elevated in vascular, infectious, and immune-mediated pathologies^11^,^12^,^13^,^14^,^15^,^16^. MPs are heterogeneous in size, composition, density, and cellular origin. MPs derived from different cell types possess unique functional capabilities due to variations of lipids and proteins acquired from parent cells^17^,^18^,^19^. The main fraction of circulating MPs in a non-diseased state is reported to be platelet or megakaryocyte-derived MPs^20^,^21^. Circulating MP in the absence of disease likely originate from aging cells^22^. Despite many studies on the role of MPs in diseases, the functional importance of normally present MPs is unclear. Circulating MPs in healthy controls were shown to support low-grade thrombin generation by the contact pathway^23^. Whether MPs originating under physiological circumstances can provide sufficient activity to support blood coagulation is not clear. Particularly little is known about potential effects of MPs on fibrin clot formation and lysis, the determinant stages of blood clotting. Fibrin is a three-dimensional filamentous network with a variable architecture that determines its properties, including chemical stability, permeability, and the ability to provide a deformable yet durable mechanical scaffold for clots and thrombi^24^. Structural features of a fibrin clot, such as the network porosity and fibre thickness, largely determine the course and outcome of hemostatic disorders^25^,^26^,^27^.

Despite various involvements of MPs in the (patho)physiology of blood clotting, there is very limited evidence for any mechanistic connections between MPs and fibrin. Addition of platelet-derived MPs to normal plasma reduced fibrin permeability^28^. Phosphatidylserine-containing artificial vesicles bound to purified fibrinogen and changed the turbidity of fibrin clots^29^. MPs generated *in vitro* bound better to plasma clots compared to fibrin clots from purified fibrinogen^30^. MPs derived from stimulated platelets and monocytes *in vitro* were shown to modulate clot formation^19^. Strong correlations between the levels of MPs, fibrin clot permeability and resistance to lysis in patients with coronary artery disease have been revealed^31^. The question remains open as to whether MPs normally present in blood have a potential to affect haemostasis and can be an additional physiological determinant of the structure and properties of a blood clot determined largely by the fibrin network scaffold. To answer this question, we studied the effects of MPs *in vitro* on the kinetics of fibrin polymerisation, fibrin network structure and susceptibility to fibrinolysis.

Here we show that MPs have significant causative effects on fibrin polymerisation and on the final structure and properties of fibrin clots. Namely, MPs support formation of dense fibrin networks composed of thin fibres resistant to enzymatic lysis via at least two mechanisms: indirectly through promoting thrombin generation and directly via interaction of MPs with fibrin(ogen). The results provide a better understanding of the mechanisms underlying formation of lysis-resistant haemostatic fibrin clots as well as clots and thrombi formed in pathological conditions associated with increased vesiculation of blood and vascular cells.

## Results

### Elimination of MPs from plasma by filtration

**Figure 1:**
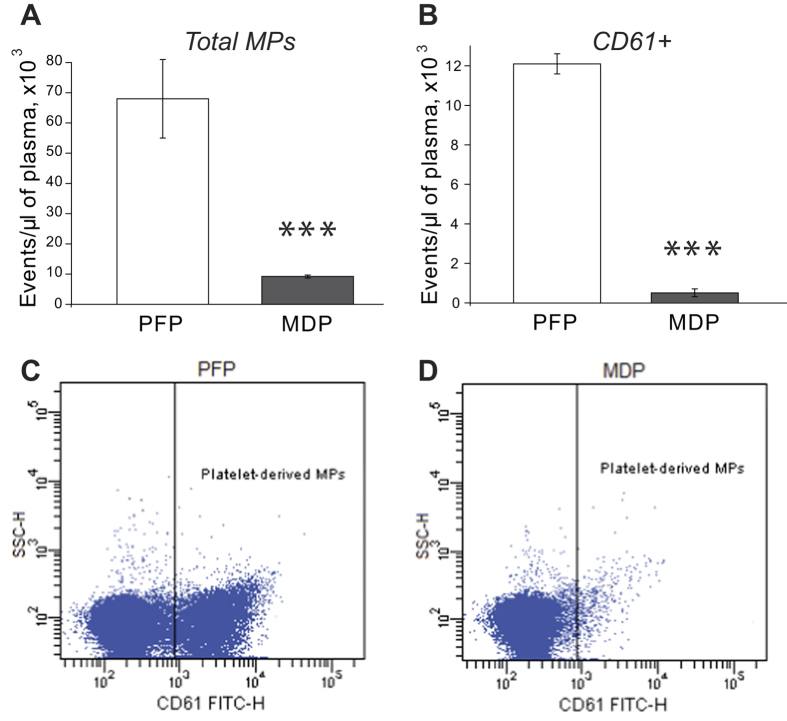
Enumeration of MPs before and after filtration of PFP. (**A**) Total MP counts before and after filtration of PFP (n = 7); (**B**) The number of CD-positive (CD61+) MPs before and after filtration of PFP (n = 7); (**C,D**) Representative flow cytometry dot-plots of CD61-positive MPs in PFP (**C**) and in the corresponding MDP (**D**). The results in A and B are presented as mean value ± SD from the measurements performed in paired plasma samples before and after filtration. Here and in all other figures PFP stands for “platelet-free plasma” and MDP stands for “microparticle-depleted plasma”.

Effects of MPs were revealed by comparing plasma samples naturally containing MPs (platelet-free plasma, PFP) and depleted of MPs (microparticle-depleted plasma, MDP) by filtration through a filter with a 0.1-μm pore size, corresponding to the lower size range of circulating MPs^1^. This approach resulted in removal of 90% of particles detectable by flow cytometry (Fig. 1A), with 99% removal of CD61+ microparticles (Fig. 1B). Fig. 1C,D show the dot-plots for platelet-derived MPs detected by the binding of anti-CD61-FITC antibodies in PFP and MDP, respectively. Importantly, average concentrations of thrombin-clottable fibrinogen in the paired PFP and MDP samples (n = 7) were found to be unchanged, 3.1 ± 0.2 g/l and 3.0 ± 0.3 g/l, respectively (p > 0.05). The average content of phospholipids (determined as the amount of lipid phosphorus) changed significantly upon plasma filtration from 2.3 ± 0.3 mmol/l in PFP to 1.1 ± 0.1 mmol/l in MDP (n = 6, p < 0.001), corroborating, in combination with the results of flow cytometry, the substantial removal of cellular membrane-derived material.

In addition, scanning electron microscopy images of 0.1-μm filters used for filtration of PFP demonstrated the presence of spherical particles, some of which were aggregated (Fig S7, *upper inset*). More than 90% of the particles retained on the filters were in the range of 80 nm to 400 nm with two peaks at 0.1 μm and ~0.3 μm (Fig S7). While MPs of this size are below the detection limit of flow cytometry, this indicates that these small MPs were also efficiently removed by filtration. Confocal microscopy of the same filters treated with FITC-labeled anti-CD61 antibodies revealed fluorescent particles having a platelet origin with a size less than 1 μm (Fig S7, *lower inset*).

### Effects of MPs on fibrin clot formation in re-calcified plasma

**Figure 2:**
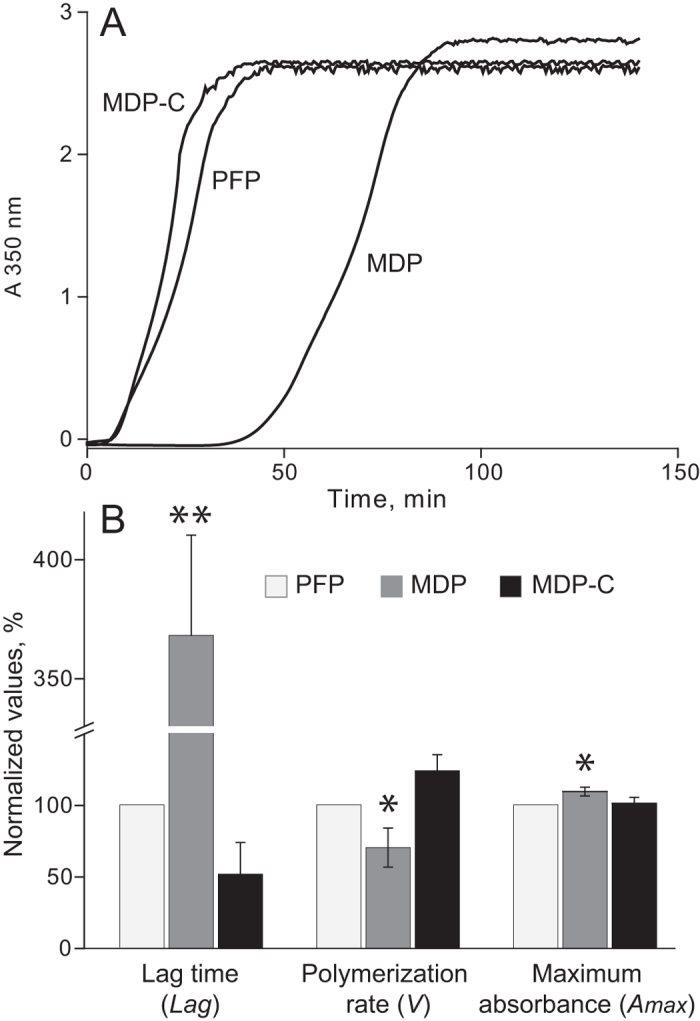
Effects of MPs on fibrin polymerisation and clot turbidity. (**A**) Representative dynamic turbidity curves obtained in PFP, MDP, and MDP-C upon re-calcification. The following parameters were determined (see Fig. S2): lag time, fibrin polymerisation rate, and maximum absorbance at the plateau. (**B**) Comparative parameters of the dynamic turbidimetry in PFP, MDP and MDP-C normalized by PFP. Since an individual experimental value for PFP in each experiment was taken as 100%, the PFP data do not have an error bar. The other results are presented as mean value ± SD from the measurements performed in three plasma variants (PFP, MDP and MDP-C) obtained from the same blood sample (n = 6). *p < 0.05, **p < 0.01 for MDP compared to PFP and MDP-C.

The effects of MPs on the kinetics of fibrin formation and on the optical properties of clots were studied using dynamic turbidimetry of re-calcified plasma samples (PFP, MDP) without adding any clotting activator (Fig. 2; Table SI). Lag time, representing the time required for thrombin generation and formation of protofibrils, was dramatically prolonged in the MDP as compared to the PFP (p < 0.01). The polymerisation rate, defined as the slope of the turbidimetric curve (Fig S2) and reflecting the lateral aggregation of protofibrils, was also significantly lower in MDP than in PFP (p < 0.05). The maximum optical density, which (for the same amount of fibrin) reflects fibre-cross-sectional area, was higher in MDP than in PFP (p < 0.05). To prove that the difference in fibrin formation was attributed to the removal of membrane-derived MPs, we replenished MDP with phospholipids (cephalin) to compensate for the loss of phospholipid-containing material removed by filtration. The amount of phospholipids added (final concentration 1.0 ± 0.1 mmol/l of lipid phosphorus) was roughly equal to the difference in phospholipid content before and after filtration of PFP. Restoration of the phospholipid levels by adding cephalin to MDP (MDP-C) caused shortening of the lag time (p < 0.01), an increase in the polymerisation rate (p < 0.05), and lowering of the maximum absorbance (p < 0.05) almost back to the base levels observed in the unfiltered PFP. These results clearly show that fibrin polymerisation and optical properties were strongly affected by the presence or absence of MPs, with phospholipids being mainly responsible for the effects of MPs.

### Effects of MPs on thrombin generation

**Figure 3:**
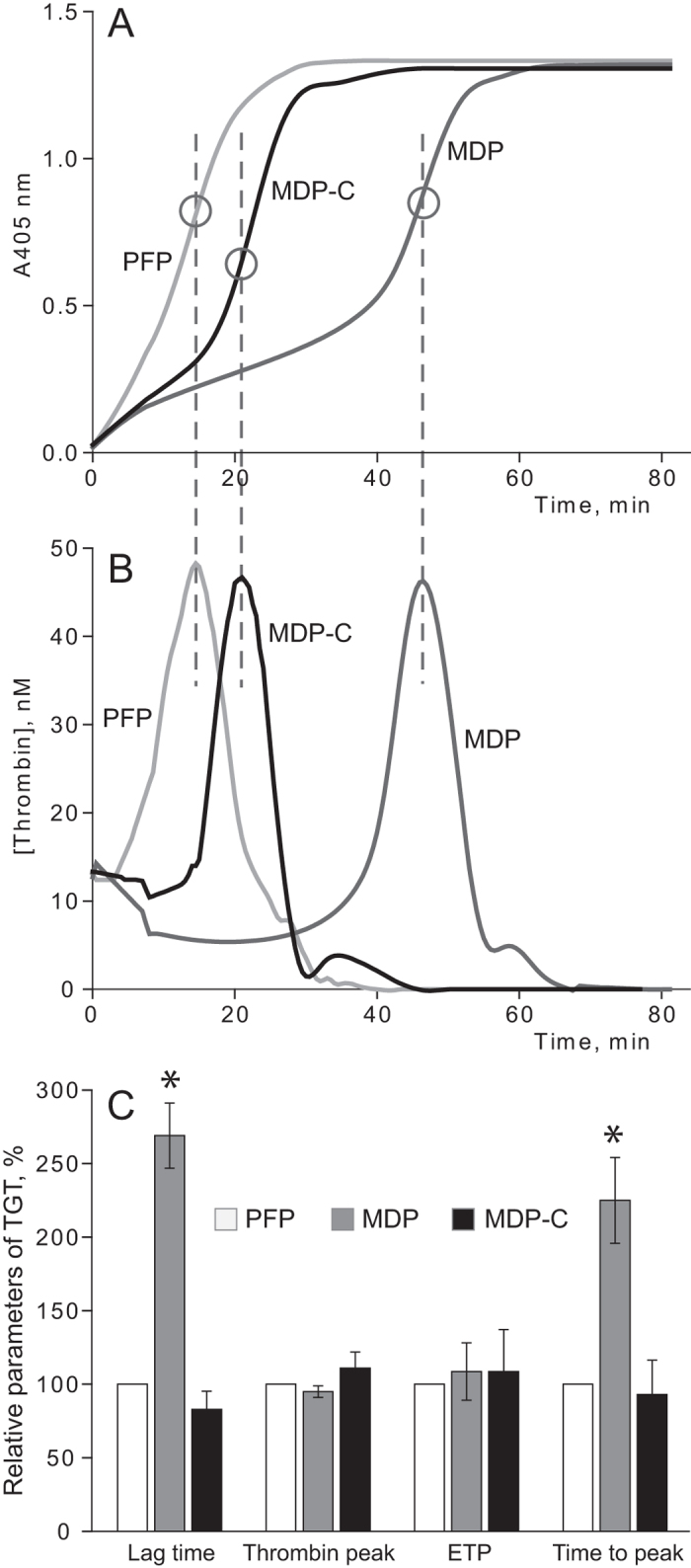
Effects of MPs on thrombin generation in plasma. (**A**) Representative colorimetric curves showing the cleavage kinetics of a thrombin chromogenic substrate (S-2238) in defibrinated PFP, MDP and MDP-C after re-calcification. (**B**) The first derivative of the colorimetric curves (“thrombograms”) corresponding to the data shown in A. (**C**) Comparative parameters of the thrombin generation test in PFP, MDP and MDP-C normalized by the values obtained in PFP. Since an individual experimental value for PFP in each experiment was taken as 100%, the PFP data do not have an error bar. The other results are presented as mean value ± SD from the measurements performed in three plasma variants (PFP, MDP and MDP-C) obtained from the same blood sample (n = 3). *p < 0.05 for MDP compared to PFP and MDP-C.

As the most likely target for MPs, thrombin generation was studied by monitoring chromogenic substrate cleavage in plasma samples before filtration (PFP), after filtration (MDP), and with addition of phospholipids to the filtered samples (MDP-C) (Fig. 3; Table SII). The Lag Time between the initiation of clotting and the start of thrombin generation (LT) and the Time required To reach Peak thrombin generation (TTP) were both prolonged in MDP (p < 0.05) compared to PFP and MDP-C, indicating that thrombin generation was reduced in the absence of MPs. On the contrary, the total amount of thrombin formed was the same in all plasma samples examined because the Peak value of Thrombin Generation (PTG) and the Endogenous Thrombin Potential (ETP) did not differ significantly in PFP, MDP and MDP-C (p > 0.05). These findings indicate that MPs contribute to fibrin polymerisation by modulating the rate of thrombin generation, not its total final activity.

### Effects of MPs on fibrin clot structure

**Figure 4:**
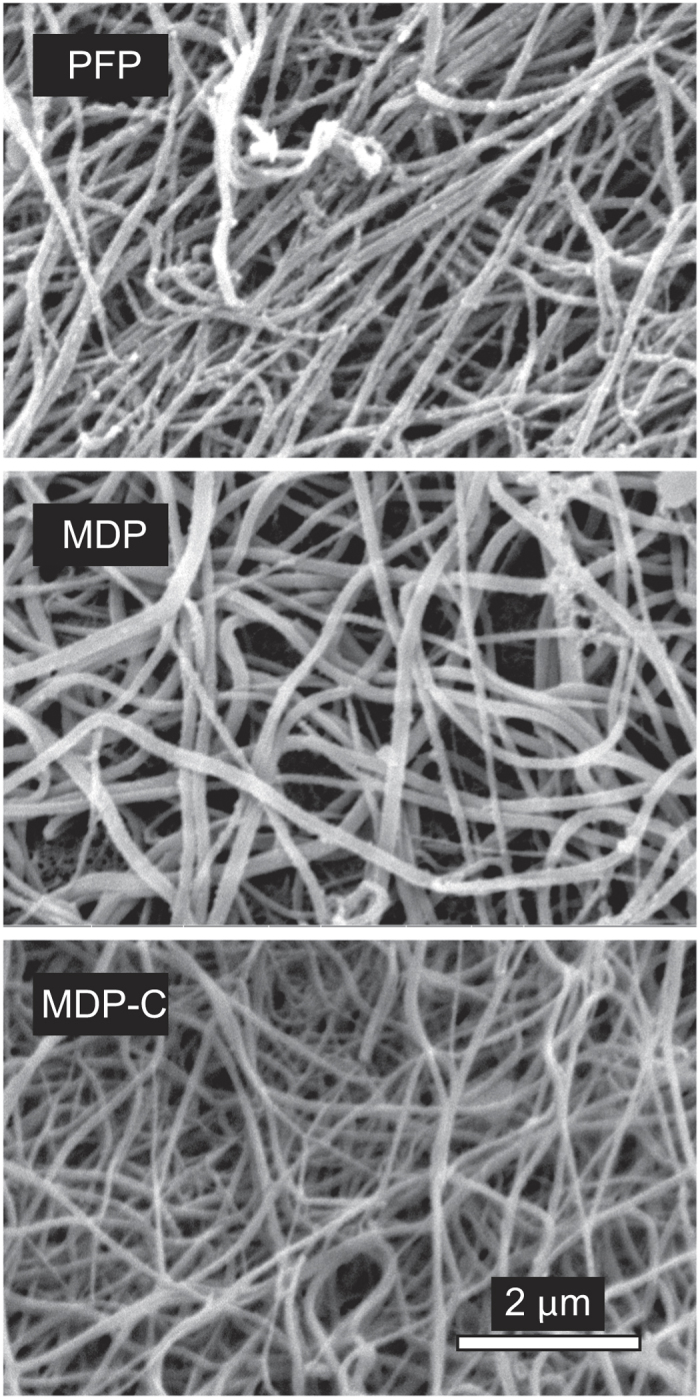
Fibrin clots ultrastructure in the presence and absence of MPs. Representative scanning electron micrographs of fibrin clots formed from the PFP, MDP, and MDP-C samples upon re-calcification. The fibrin structure in PFP and MDP-C samples is more compact with smaller intrinsic pores. The fibres in the PFP and MDP-C samples are thinner with increased branch-points and the PFP-clot also contains particulate matter on the surface of fibres. The magnification bar is 2 μm for all the images. Three individual clots were studied using scanning electron microscopy for each of the plasma sample types, PFP, MDP, and MDP-C.

Scanning electron microscopy revealed structural differences in fibrin clots made from PFP, MDP and MDP-C (Fig. 4). Clots formed in PFP were apparently more dense and had thinner fibres (170 ± 38 nm), while clots from MDP had larger pores and thicker fibres (214 ± 53 nm, p < 0.05). Addition of phospholipids to MDP (MDP-C) resulted in the formation of densely packed thin fibrin fibres (141 ± 34 nm), similar to the initial PFP-clots. In addition, the fibrin fibres formed in MDP were quite smooth, while fibres formed in the presence of MPs (PFP) or exogenous phospholipids (MDP-C) had rough surfaces with multiple sub-micron-size particles attached to the fibres, suggesting that phospholipids might directly bind fibrin.

### Effects of MPs on internal fibrinolysis

**Figure 5:**
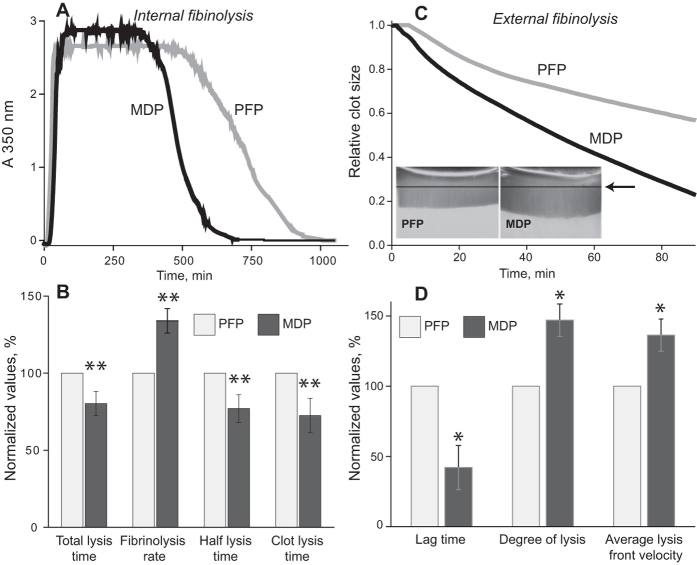
Effects of MPs on internal (A,B) and external (C,D) fibrinolysis. (**A**) Representative clot lysis curves in PFP (grey line) and the corresponding MDP (black line). Clots were lysed by adding equal amounts of t-PA to the plasma samples before clotting was initiated. (**B**) Comparative parameters of internal fibrinolysis (see Fig. S5) in PFP and MDP normalized by PFP. (**C**) Time course of the directional clot lysis corresponding to the inserted images of the lysis front taken 90 minutes after loading of t-PA on top of the pre-formed PFP- and MDP-clots. The arrow points to the initial clot levels. (**D**) Comparative parameters of external fibrinolysis (see Fig. S6) in PFP and MDP normalized by PFP. Since an individual experimental value for PFP in each experiment was taken as 100%, the PFP data do not have an error bar. The other results are presented as mean value ± SD from the measurements performed in 4 paired plasma samples for each type of fibrinolysis. *p < 0.05, **p < 0.01.

Clot formation and lysis were followed by the dynamic turbidity of re-calcified plasma to which t-PA was added (Fig. 5A,B; Table SIII). The amount of t-PA was adjusted to trigger fibrinolysis after the absorbance reached a plateau to ensure that the polymerisation was complete and that t-PA did not affect clot formation. All the parameters characterising the clot lysis time and the lysis rate differ significantly in PFP and MDP (p < 0.01). The results indicate that fibrin clots formed in the presence of MPs are less susceptible to the t-PA-induced internal fibrinolysis.

### Effects of MPs on external fibrinolysis

External fibrinolysis is complementary to internal fibrinolysis and reproduces thrombolytic therapy with a lytic enzyme acting from outside a clot. Continuous imaging of clots pre-formed from re-calcified PFP and MDP was used for real-time assessment of the external fibrinolysis initiated by a t-PA solution placed on one side of the clots. The lysis progressed as a sharp front moving down from the top of the clots where t-PA was applied. The lysis front velocity was significantly lower in the PFP- compared to MDP-clots (Fig. 5C, *inset*). Quantification of the kinetics curves (Fig. 5C,D; Table SIV) revealed that the lag time was shorter in MDP-clots compared to PFP-clots (p < 0.05). Both the lysis front velocity and the degree of lysis were higher in MDP-clots than in PFP-clots (p < 0.05). In agreement with the effects of MPs on the internal lysis, these results demonstrate that plasma clots containing MPs are more resistant to externally applied fibrinolytics than clots formed in the absence of MPs.

### Effects of MPs on fibrin clot formation induced by exogenous thrombin

To rule out an indirect kinetic effect of MPs on fibrin formation due to their ability to accelerate generation of the endogenous thrombin in re-calcified plasma, fibrin formation was induced directly by addition of the exogenous thrombin in the absence of Ca^2+^. The effect of MPs on thrombin-induced fibrin formation was evaluated by comparing turbidimetric curves obtained in the unfiltered (PFP) and filtered (MDP) plasma samples as well as in the same filtered plasma sample replenished with cephalin (MDP-C) (Fig S8). There was no significant difference in the polymerisation rate. However, the maximum optical density was moderately but consistently higher in MDP compared to PFP and MDP-C (p < 0.05). The results suggest that the final clot structure is directly affected by the presence or absence of MPs, irrespective to their ability to accelerate thrombin generation. The possibility that MPs directly interact with fibrin is strongly confirmed by about a 2-fold reduction of the average number of MPs in serum after removal of clots from the thrombin-activated initial PFP samples (39,035 ± 7,947/μl vs. 70,528 ± 10,679/μl, respectively; n = 6, p < 0.001).

### Attachment of MPs to fibrin fibres

**Figure 6:**
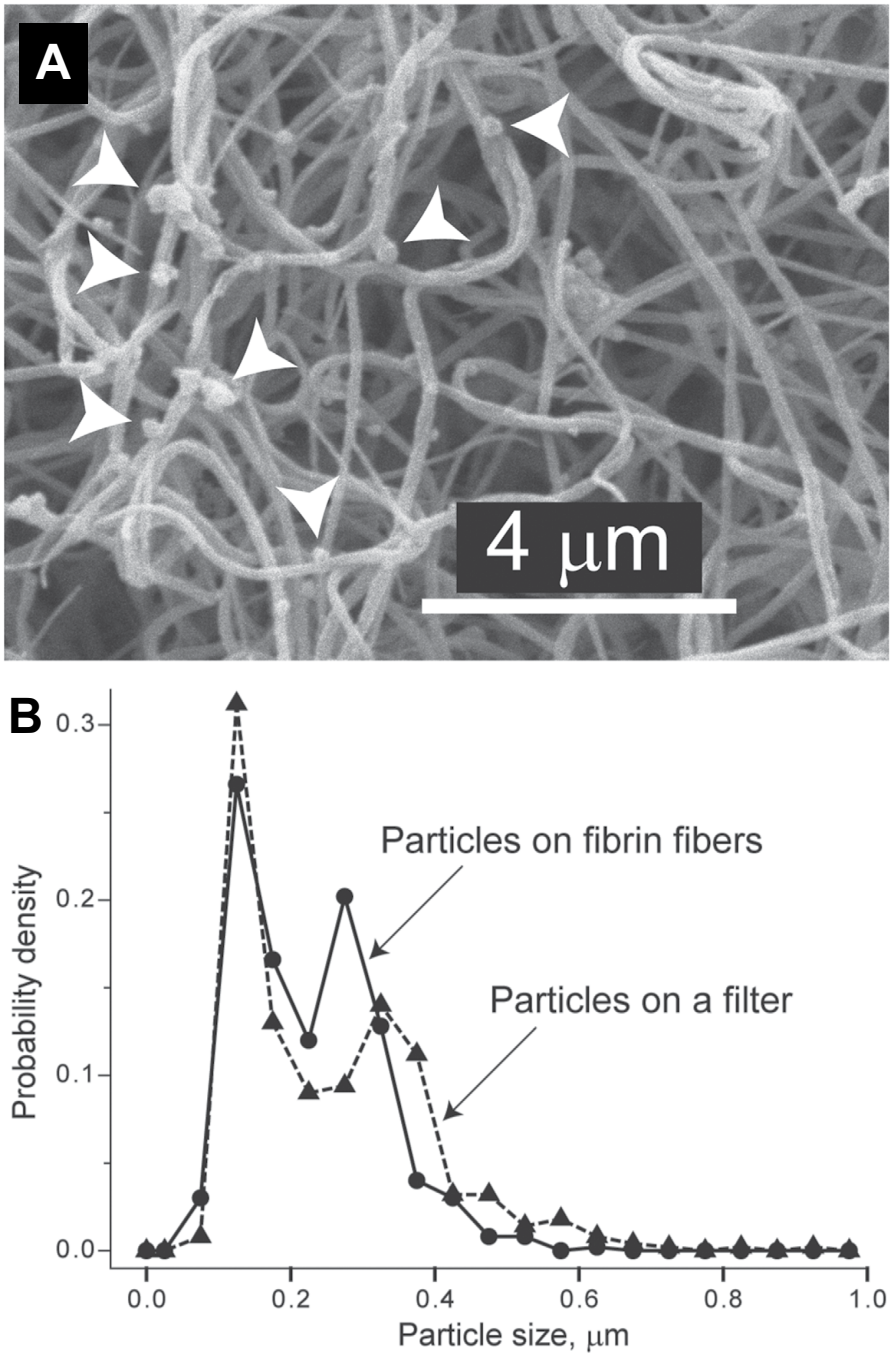
Direct visualization of microparticle-like structures on fibrin fibres. (**A**) Scanning electron microscopy showing grainy particles (white arrows), likely to be MPs, on the fibres of a fibrin clot prepared from re-calcified PFP. (**B**) Normalized size distributions (histograms with a 0.05-μm bin width) for the particles (n = 500) visualized on a 0.1-μm-pore size filter after filtration of PFP (see Fig. 1, upper inset) and for the particles (n = 500) detected on fibrin fibres obtained from PFP.

**Figure 7:**
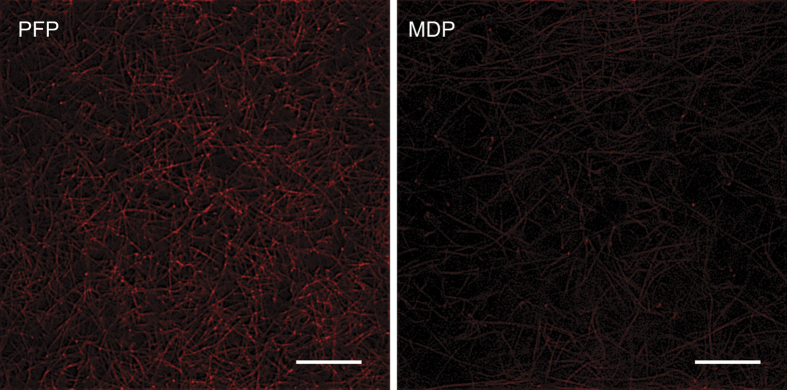
Visualization of platelet-derived microparticles on fibrin fibres. Representative confocal microscopy images of clots obtained from PFP (left) vs. MDP and stained for CD61 with Alexa 647-labeled antibodies (n = 6). The images were adjusted to the same levels of brightness and contrast to make them comparable. Unlike the MDP clot, the PFP clot had significantly higher fluorescence intensity associated with the fibrin network, some of which is present as grainy spots, demonstrating binding of platelet-derived material to fibrin fibres. The magnification bar is 20 μm.

Scanning electron microscopy images of thrombin-induced clots differed in that fibrin fibres formed after depletion of MPs (MDP) were smooth, while fibrin fibres formed in the presence of MPs (PFP) or exogenous phospholipids (MDP-C) had sub-micron-size structures attached to the fibres (Fig S9). This was similar to what was observed on fibrin fibres obtained upon re-calcification of PFP and MDP-C vs. MDP (Fig. 4). Notably, the size of the fibrin-attached particles varied within 0.1–0.5 μm with two characteristic peaks at 0.1 μm and 0.3 μm, which turned out to be quite similar to the size distribution of the vesicles revealed on a filter used to remove MPs from PFP (compare Fig. 6 and S7). The results strongly suggest that the particles attached to the fibrin fibres in the PFP-clots likely represent MPs. To confirm the platelet origin of the fibrin-bound particles, the PFP and MDP clots were stained with Alexa-647-labeled anti-CD61 antibodies and analyzed by confocal microscopy (Fig. 7). The clots made of PFP demonstrated higher fluorescence intensity arranged in small, discrete spots and associated with the fibrin network, indicating that platelet-derived material was adsorbed on fibrin fibres. Much weaker fluorescence with rare spots was revealed in MDP-clots due to much fewer platelet-derived MPs (Fig. 1). Fibrin clots formed in PRP were used as a positive control for platelet-associated specificity of the labeled anti-CD61 antibodies (Fig. S10).

## Discussion

The role of MPs in haemostasis and thrombosis has been a hot topic over the past decades^10^,^32^,^33^,^34^,^35^. Despite numerous studies on the association of MPs with various pathological conditions, it is still unclear whether circulating MPs present in the blood of healthy subjects may affect blood clotting and haemostasis, including their impact on the formation and properties of fibrin networks, a major structure and a mechanical scaffold of clots and thrombi. The only direct observation on the role of platelet- and monocyte-derived MPs in fibrin formation was performed using MPs obtained *ex vivo* from stimulated cells^19^, which might be different from MPs formed *in vivo* by composition and properties. Whether or not the blood of healthy individuals contains sufficient levels of functional MPs to influence the properties of fibrin clots remained unclear until now.

To determine the importance of circulating MPs we used an approach based on elimination of MPs from normal platelet-free plasma (PFP) by filtration, yielding microparticle-depleted plasma (MDP). MPs were removed with a 0.1-μm-pore filter, which corresponds to the conventional lower limit of MP size^1^,^36^, although the range of MP dimensions remains disputable^37^. A similar filtration-based approach was used earlier in a study of cell-derived vesicles and exosomes^38^. MPs in plasma before and after filtration were measured using size-based flow cytometry. Although this method perhaps misses a substantial fraction of smaller MPs^39^, nevertheless it is informative and is the most commonly used approach. Combined analysis of PFP and MDP by flow cytometry and the particles on filters by confocal and scanning electron microscopy showed that >90% of MPs were eliminated during filtration, including >99% of platelet- and/or megakaryocyte-derived MPs (Fig. 1), which are the most abundant fraction of MPs in healthy individuals^20^,^23^. It is very important to note that the amount of clottable fibrinogen in plasma did not change after the filtration, which rules out the possibility that the observed differences between PFP and MDP could result from potential elimination of fibrinogen and its derivatives. Complete restoration of thrombin generation after replenishment of MDP with phospholipids (Fig. 3) confirms that other coagulation factors were not affected by filtration. This finding is in agreement with the work showing that filtration of plasma through a 75-nm nanofilter fully preserves protein and lipoprotein profile, coagulation factor content, and global coagulation activity (prothrombin time, activated partial thromboplastin time) despite an extensive removal of MPs^40^. Lipoprotein composition of MDP also remained unchanged after filtration in our experiments.

This study shows that MPs existing in the blood of apparently healthy individuals have profound effects on fibrin formation, the final network structure and stability of fibrin clots. First of all, we examined the overall effect of MP removal on fibrin clot growth kinetics in re-calcified plasma samples. A dramatic decrease in the time required for thrombin generation and fibrin polymerisation was seen in MDP vs. PFP, i.e. in the absence and presence of MPs, respectively (Fig. 2; Table SI). The turbidity profile of clot formation in MDP was fully recovered after equivalent reconstitution of phospholipids, corroborating the argument that the observed effects were associated with MPs. Because there was no exogenous clotting activator used, the most likely underlying mechanism for the observed changes was different thrombin generation determined by the procoagulant phospholipid component of MPs, phosphatidylserine and/or some other mechanisms. This assumption was confirmed by studying thrombin generation in plasma samples before and after removal of MPs and with addition of phospholipids to MDP. Expectedly, thrombin generation was dramatically delayed in MDP compared to PFP and MDP-C, but the total amount of thrombin generated was the same in all plasma samples (Fig. 3; Table SII), indicating that MPs contribute to fibrin polymerisation and clot structure indirectly by modulating the time course of thrombin generation, not its total final activity. Our results are consistent with the finding showing that the presence of tissue factor-expressing MPs in plasma shortened the lag time but did not change the overall thrombin generation^41^.

To elucidate the differences of the final turbidity of re-calcified clots in the presence and absence of MPs, we studied the ultrastructure of the clots by scanning electron microscopy. In the presence of MPs or exogenous phospholipids, the fibrin networks had less porous structures with thinner fibres (Fig. 4), in agreement with the earlier finding that lower turbidity is correlated with thinner fibrin fibres^42^. The effect of MPs on the structure of the fibrin network is apparently related to the ability of MPs to accelerate thrombin formation because thrombin activity is known to strongly influence fibrin structure in just such a manner. In particular, a high thrombin activity results in a fine network of thin fibres whereas fibrin polymerisation at a lower thrombin activity results in clots composed of thicker fibres due to slower elongation and greater lateral aggregation of protofobrils^27^.

The effects of MPs on fibrin structure must have functional consequences and change clot properties, including susceptibility to fibrinolytic proteases. The effects of MPs on t-PA-induced fibrinolysis were monitored in two complementary model systems, namely, internal and external fibrinolysis *in vitro*. The former model mimics natural dissolution of a hemostatic clot *in vivo*, while the latter model reproduces thrombolytic therapy. Consistent with the dissimilarity in the network structure, clots formed by re-calcification in the presence of MPs (PFP) were significantly more resistant to both internal and external lysis (Fig. 5; Tables SIII and SIV). These results are in line with the observation that the number of fibres per volume determines resistance to the enzymatic lysis rather than an individual fibre diameter^43^. The delay in fibrinolysis rates in densely packed clots with thinner fibres is likely due to the greater number of fibres in the clot that need to be cleaved and other differences in spatio-temporal protein distributions^44^,^45^. There are a number of other factors that influence lysis speeds in clots of varying structure^46^. To avoid artifacts due to direct influence of phospholipids on the activity of fibrinolytic enzymes^47^ exogenous phospholipids were not added to MDP in the experiments with internal and external fibrinolysis.

Thus, we demonstrated that circulating MPs affect all stages of fibrin polymerisation kinetically by increasing the rate of thrombin generation, which leads to the formation of denser fibrin clots with thinner fibres that are less susceptible to fibrinolysis. However, the indirect kinetic influence of MPs on fibrin may not be the only mechanism. A conceivable alternative way to modify clots could be direct physical interaction of MPs with fibrin because various lipids, including phospholipids, have been shown to bind fibrin(ogen) and exert marked influence on architecture, mechanical properties, and stability of clots^28^,^29^,^47^,^48^,^49^.

To exclude the indirect kinetic effects of MPs on fibrin via endogenous thrombin, fibrin formation was induced by adding exogenous thrombin without Ca^2+^ (to prevent formation of endogenous thrombin) in the absence and presence of MPs. Under these experimental conditions, we bypassed thrombin generation on phospholipids, and fibrin formation occurred in response to an unvarying thrombin activity. Turbidimetry revealed that in the presence of natural MPs (PFP) or exogenous phospholipids (MDP-C) the maximal turbidity of the fibrin clot was significantly smaller than in the absence of MPs (MDP) (Fig. S8). Since the level of MPs was the only variable, this result suggests that the observed changes in the clot optical properties and structure result from direct interaction of MPs with fibrin. This presumption is strongly confirmed by a 45% reduction in the MP count after clotting of PFP followed by physical removal of the clot.

Perhaps the most powerful argument for the physical interaction of MPs with fibrin is the existence of sub-micron-size particles associated with fibrin fibres formed in the presence of MPs, while fibrin fibres formed without MPs were smooth and not coated with any grainy matter (Fig. 4, S9). Importantly, the size distribution of these fibrin-attached particles is almost identical to the size histogram of the vesicles found on filters used for the removal of MPs from plasma (Fig. 6), which strongly suggests direct incorporation of MPs into the fibrin network. The observed size of the granular material, which we identify as circulating MPs, corresponds to the heterogeneous dimensions of cell-derived MPs reported by others^50^,^51^,^52^. Consistent with the results of scanning electron microscopy, confocal microscopy of PFP-clots stained for CD61 revealed the presence of platelet-derived material on fibrin fibres (Fig. 7). Binding of platelet-derived MPs to fibrin can be mediated by direct adsorption of phospholipids^48^,^53^. It could be also mediated by the active platelet integrin αIIbβ3 which has a strong affinity for fibrin(ogen)^54^. This integrin has been recently shown to reside on erythrocytes^55^; therefore, it can potentially anchor erythrocyte-derived MPs to fibrin as well. In addition to the integrin-mediated adhesion, MPs can be embedded into a fibrin clot via electrostatic and hydrophobic interactions^53^. It is likely that MPs affect fibrin formation and structure by interfering with the lateral aggregation of protofibrils, but the mechanism and physiological consequences of the direct interactions between MPs and fibrin found in this study certainly need further investigation.

Irrespective of the underlying mechanisms, our results show that circulating MPs support formation of more compact fibrin clots that are less susceptible to fibrinolysis. This finding may have physiological and clinical significance because MPs turn out to be an additional and so far underappreciated modulator of the formation and properties of a hemostatic clot. Even normally circulating MPs can support the formation of stable clots with a prolonged lifetime at the sites of vascular injury. In pathological conditions associated with an augmented formation of MPs, the pathogenic role of MPs can be much more important because their massive incorporation into arterial or venous clots and thrombi is a potential cause of ineffective fibrinolysis and therapeutic thrombolysis.

In conclusion, normally circulating MPs have profound effects on fibrin formation and on the final structure and characteristics of fibrin clots via at least two mechanisms. One is an indirect kinetic effect based on the MP-dependent rate of thrombin generation. The other is direct binding of MPs to fibrin fibres during and after fibrin assembly. Both mechanisms underlie a previously underappreciated potential role of MPs in haemostasis and thrombosis as modulators of fibrin formation and properties.

## Methods

### Platelet-free plasma (PFP) and microparticle-depleted plasma (MDP)

Blood was obtained from healthy volunteers (average 26 ± 5-year-old) not taking aspirin, nonsteroidal antiinflammatory drugs, or other medications known to affect clotting factors or platelet function for at least 7–10 days, with informed consent from all subjects and approval by the Ethical Committee of Kazan State Medical University. All procedures were carried out in accordance with the approved guidelines. Blood was drawn via venipuncture into 3.2% trisodium citrate (9:1). Immediately after collection, citrated blood was centrifuged at room temperature at 1,500 g for 15 min followed by centrifugation of the supernatant at 10,000 g for 5 min to obtain PFP. A portion of PFP was filtered through a 0.1-μm-pore size filter (SLVV033RS, EMD Millipore, Billerica, MA) to remove MPs and collect MDP. To restore the phospholipids eliminated by filtration, a portion of MDP was replenished with a suspension in PBS of phosphatidylethanolamine and phosphatidylserine known as cephalin (MDP-C) at a final concentration of 300 μg/ml equivalent to ~1 mmol/l of lipid phosphorus. Fresh samples of PFP, MDP, and MDP-C from the same donors (n = 55) were analyzed in parallel.

### Characterization of MPs using flow cytometry

To quantify MPs in plasma before and after filtration and in the corresponding serum samples a FACSCalibur flow cytometer was used^56^. 10,000 total events were collected during each sample analysis using the forward (FSC) versus sideward (SSC) scatter dot plots. To gate for MPs, the upper size limit of 1 μm was set using beads of a known diameter (Fig. S1 – Supplementary Material). To quantify platelet-derived MPs in PFP and MDP, the expression of platelet-specific antigen CD61 was studied using mouse anti-human-CD61 FITC-antibodies and respective isotype-matched control antibodies (BD Biosciences, San Jose, CA). Because phospholipids comprise a major component of MPs, the content of endogenous phospholipids in PFP, MDP and the amount of phospholipids added to MDP were determined according to Gent *et al.*^57^.

### Dynamic turbidimetry of plasma clot formation

Clotting of PFP, MDP or MDP-C samples induced by either Ca^2+^ (24 mmol/l) or α-thrombin (HYPHEN Biomed, France; 0.1 U/ml equivalent to 1.2 nmol/l) was followed by monitoring the optical density at λ = 350 nm at 37 °C using a Perkin-Elmer Lambda-25 spectrophotometer (Molecular Devices, Sunnyvale, CA). The extracted parameters of fibrin formation are shown in Fig. S2.

### Thrombin generation assay

The amount of thrombin formed in plasma upon re-calcification was measured directly using a modified thrombin generation test^58^,^59^,^60^. Because fibrin interferes with colorimetric measurements, PFP and MDP samples were first defibrinated by adding reptilase (Sekisui Diagnostics, Lexington, MA; 7.5 mg/ml) followed by incubation for 30 min at 37 °C. The clots were removed by winding them on a plastic spatula. Then a chromogenic substrate for thrombin S-2238 (Chromogenix, 2 mmol/l) was added to the plasma samples. Thrombin generation was started by adding CaCl_2_ (24 mmol/l) with simultaneous recording of the absorbance at λ = 405 nm. The first derivative of the dynamic optical density was taken to yield a thrombin generation curve (“thrombogram”) and to extract numerical parameters (Figs S3 and S4).

To assess if defibrination of plasma affects the content of MPs, we quantified MPs in PFP and MDP using flow cytometry before and after clot formation followed by clot removal. In both samples the reduction in average MP counts after clot removal was roughly equal, namely ~45% for PFP and ~50% for MDP. Therefore, the defibrination itself could not distort the comparative effects of MPs on thrombin generation.

### Internal fibrinolysis

Internal fibrinolysis was studied by mixing PFP or MDP with CaCl_2_ (24 mmol/l) to initiate fibrin formation and t-PA (HYPHEN BioMed; 50 ng/ml) to activate lysis of the clot from inside. Fibrin polymerisation and dissolution were monitored by a Perkin-Elmer Lambda-25 spectrophotometer at λ = 350 nm. The lysis rate was characterized by the parameters shown in Fig. S5.

### External fibrinolysis

External fibrinolysis was studied by continuous photographic monitoring of a fibrin clot during its dissolution from one side. PFP- or MDP-clots were pre-formed from re-calcified plasma samples in a transparent chamber. Lysis was initiated by 10 μl of t-PA (1 μg/μl) applied on the top of a clot about 12 × 7 × 1 mm in size formed from 90 μl of plasma in a plastic cuvette. Propagation of the lysis front was detected by taking frontal digitized images every 60 s over 90 min (the relatively fast lysis phase) followed by a computational analysis that generated kinetic curves characterized by a number of parameters (Fig. S6).

### Scanning electron microscopy of fibrin clots and filters

Fibrin clots from PFP, MDP or MDP-C were formed at 37 °C for 2 hrs by adding CaCl_2_ (24 mmol/l), washed in PBS (3 × 15 min), then fixed in 2% glutaraldehyde, dehydrated, dried with hexamethyldisilazane, and sputter-coated with gold-palladium. Fibre thickness was measured using the ImageJ software in randomly selected areas for a total of 200 fibres per clot (3 clots per each experimental condition). Polycarbonate filters with 0.1-μm pore size (EMD Millipore) after filtration of PFP were prepared for the scanning electron microscopy as described. The samples were examined in an FEI Quanta 250 scanning electron microscope (FEI, Hillsboro, OR).

### Fluorescent confocal microscopy of fibrin clots and filters

Fibrin clots were visualized by fluorescent confocal microscopy after clotting of platelet-rich plasma (PRP), PFP, or MDP with CaCl_2_ (24 mmol/l) at 37 °C for 2 hrs. The clots were treated with Alexa- 647-labeled mouse anti-human-CD61 antibodies (BioLegend, San Diego, CA) and analyzed in the confocal microscope. Filters used for filtration of PFP were immersed into a buffer containing FITC-anti-CD61 antibodies followed by confocal microscopy. The same clots and filters treated with isotype-matched antibodies showed no fluorescence. The imaging was performed with Confocal Laser Scanning System LSM780 (Carl Zeiss, Germany) with 2 lasers, an argon ion laser (488-nm excitation wavelength and BP 505–530-nm filter for the FITC label) and a helium-neon laser (633-nm excitation wavelength and LP 655-nm filter for the Alexa 647 label).

### Statistical analyses

All experimental data were analyzed by the paired Student’s *t*-test using the Microsoft Excel software. Data are shown as means and standard deviations (SD), and *P* values are reported as follows: *P < 0.05, **P < 0.01, and ***P < 0.001.

## Additional Information

**How to cite this article**: Zubairova, L. D. *et al.* Circulating Microparticles Alter Formation, Structure, and Properties of Fibrin Clots. *Sci. Rep.* **5**, 17611; doi: 10.1038/srep17611 (2015).

## Supplementary Material

Supplementary Information
