# Supplementary material for: Circulating Microparticles Alter Formation, Structure, and Properties of Fibrin Clots
Source: Sci Rep. 2015 Dec 4;5:17611. doi: 10.1038/srep17611 (PMC4669434; doi:10.1038/srep17611)
Supplement: Supplementary Information [file srep17611-s1.pdf]

Supplementary Information

Circulating Microparticles Alter Formation, Structure, and Properties of Fibrin Clots

Laily D. Zubairova[1], Roza M. Nabiullina[1], Chandrasekaran Nagaswami[2], Yuriy F. Zuev[3], Ilshat G. Mustafin[1], Rustem I. Litvinov[2,4], John W. Weisel[2,*]

[1]Department of General Pathology, Kazan State Medical University, Kazan 420012, Russian Federation
[2]Department of Cell and Developmental Biology, University of Pennsylvania School of Medicine, Philadelphia, Pennsylvania 19104, USA
[3]Institute of Biochemistry and Biophysics, Russian Academy of Sciences, Kazan 420111, Russian Federation
[4]Institute of Fundamental Medicine and Biology, Kazan Federal University, Kazan 420012, Russian Federation

Supplementary Figures

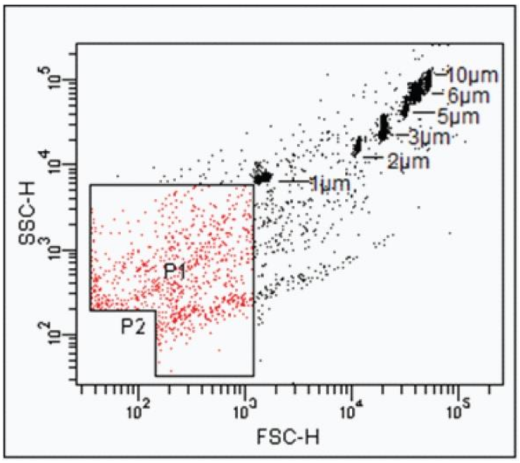

Figure S1. Flow cytometry gating for particles <1 μm using size-based calibration with synthetic beads 1 μm, 2 μm, 3 μm, 5 μm, 6 μm, and 10 μm in diameter. P1 represents the region for counting MPs, P2 represents the background noise excluded from the count.

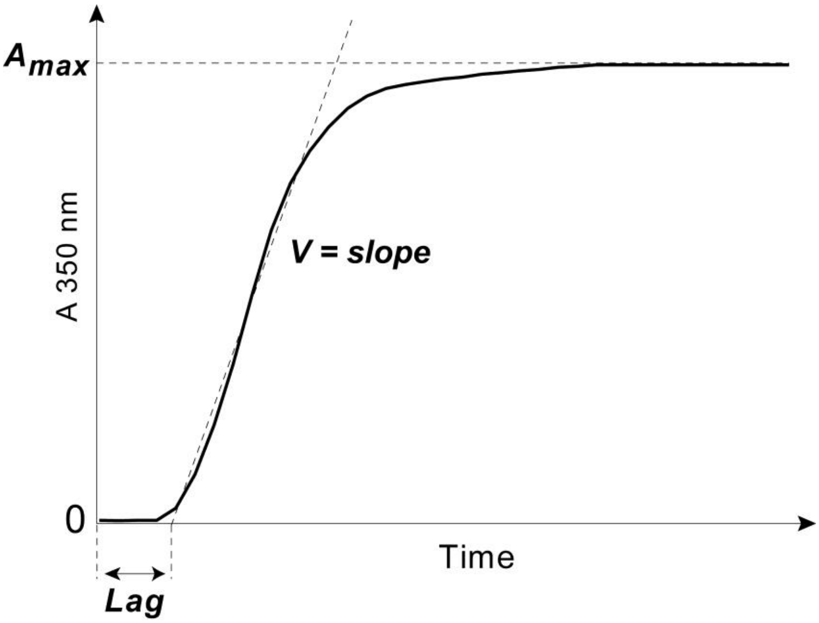

Figure S2. A typical turbidimetry curve with the following extracted parameters of fibrin formation: 1) the lag phase (Lag) or the time from adding $CaCl_2$ until an increase of the optical density by 0.01, which measures the time needed for thrombin generation and protofibril formation; 2) the slope of the curve or the rate of polymerisation (V) taken between the end of the lag phase through the linear part of the curve, which measures the velocity of lateral aggregation of protofibrils and fibre formation; 3) the maximum optical density (A_{max}) at the plateau, which reflects the amount of fibrin formed and the fibrin fibre cross-sectional area.

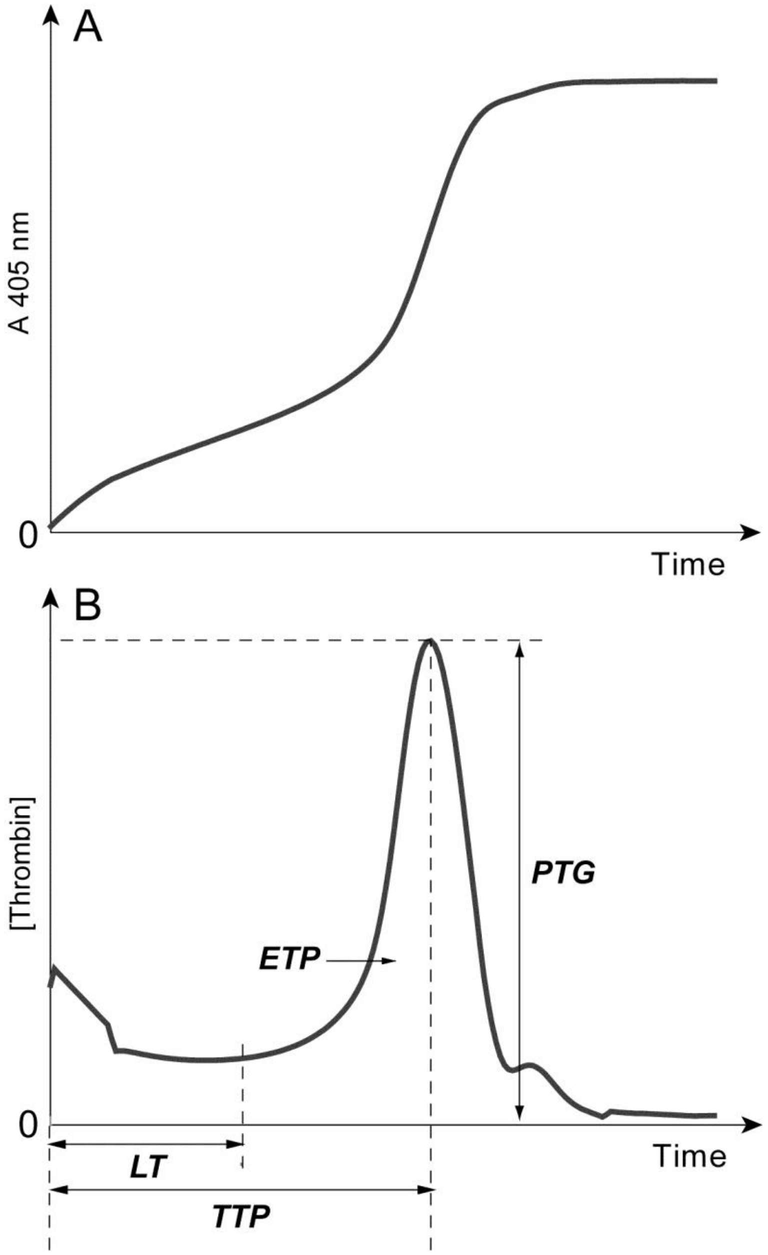

Figure S3. Thrombin generation assay. (**A**) A sample of raw photometric curve reflecting the time-dependent cleavage of a thrombin-specific chromogenic substrate in a re-calcified plasma sample. (**B**) The first derivative of the curve shown in A, i.e. a curve similar to the “thrombogram” [Hemker et al., *Pathophysiol. Haemost. Thromb.*, 2003, 33, 4–15] that enabled us to extract the following parameters of thrombin formation: 1) lag time (LT), i.e. the time from the initiation of clotting to the beginning of a stable increase of the first derivative, reflecting an increase in the optical density due to cleavage of the chromogenic substrate; 2) Time To the Peak of thrombin generation (TTP), i.e., the time required to reach maximum thrombin concentration; 3) Peak value of Thrombin Generation (PTG), i.e., the highest thrombin concentration; and 4) the Endogenous Thrombin Potential (ETP), i.e., the area under the thrombin generation curve.

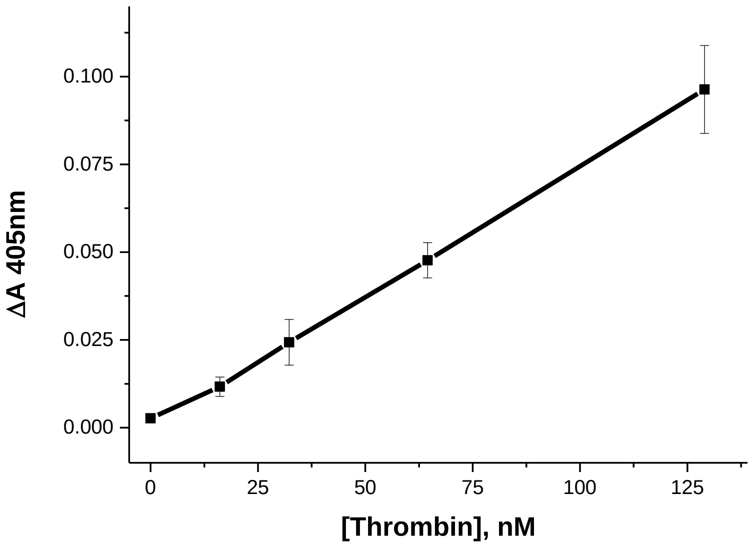

Figure S4. Thrombin generation assay. A reference curve built to convert the dynamic optical density of the chromogenic substrate cleaved in plasma into absolute thrombin concentration, which was prepared using purified human thrombin. Certain amounts of purified human thrombin (0; 0.625; 1.25; 2.5; 5 NIH Units/mL) (HYPHEN BioMed) were added to defibrinated plasma containing 10 mM S-2238. The absorbance at 405 nm was recorded. NIH units were converted into a molar concentration of α-thrombin based on the molecular weight (37 kDa) and the fact that 4.35 µg (0.12 nmol) of the thrombin preparation had 10 NIH units activity.

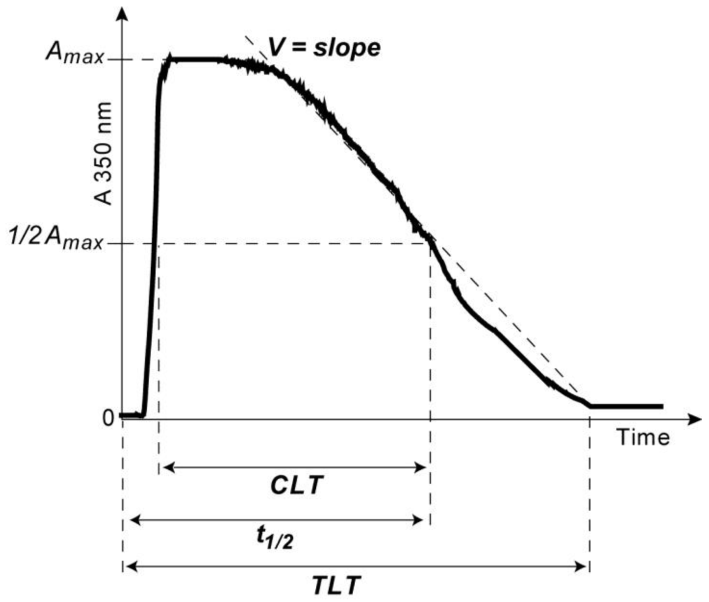

Figure S5. A typical turbidimetry curve of fibrin formation and lysis with the following extracted parameters of the intrinsic fibrinolysis: 1) Total Lysis Time (TLT), i.e., the time from adding $CaCl_2$ and t-PA until the time point when the OD almost reaches the baseline and not changes any more, which corresponds to complete clot dissolution; 2) the rate of fibrin lysis (V), i.e., the slope of the descending limb of the curve between the end of the horizontal plateau and the end point of TLT; 3) the time needed to reduce the maximum turbidity of the clot to the half-maximal value ($t_{1/2}$); 4) Clot Lysis Time (CLT), which was defined as the time from the midpoint of the clear to maximum turbid transition, which characterizes clot formation, to the midpoint of the maximum turbid to clear transition, which represents clot lysis [Lisman T., et al., *Blood*, 2005, 105(3), 1102-1105].

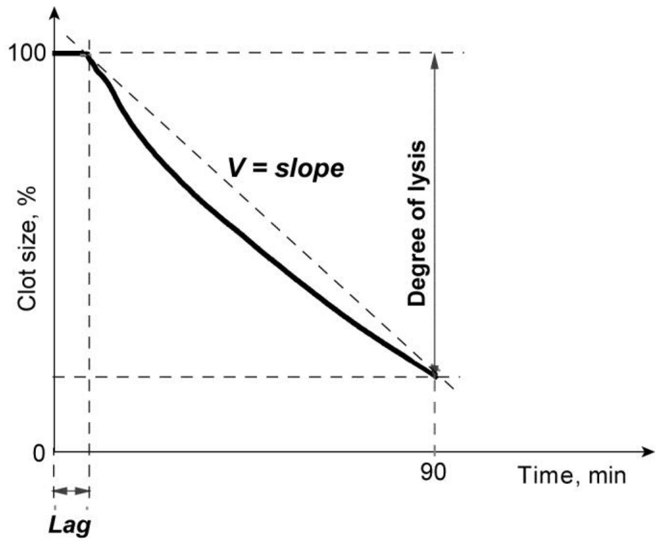

Figure S6. A typical kinetic curve, reflecting propagation of the clot lysis front with the following extracted parameters of the external fibrinolysis: 1) lag time (Lag), the time in minutes from the addition of t-PA to the first signs of fibrinolysis reflected by decrease in the initial optical density by 5%; it means the time needed for formation of t-PA/plasminogen/fibrin complexes and plasminogen to plasmin conversion; 2) degree of lysis, reflecting the difference of initial and final clot size after 90 min (in %); this time point was defined empirically as the end of the phase of fast lysis in which the differences in the lysis rate, if they exist, become clear; 3) the slope of the curve (V) or an average lysis front velocity, defined as a difference in % of initial and final clot size per time. *Note*: because the lysis front is not linear, the rate of lysis is followed along the middle line perpendicular to the lysis front.

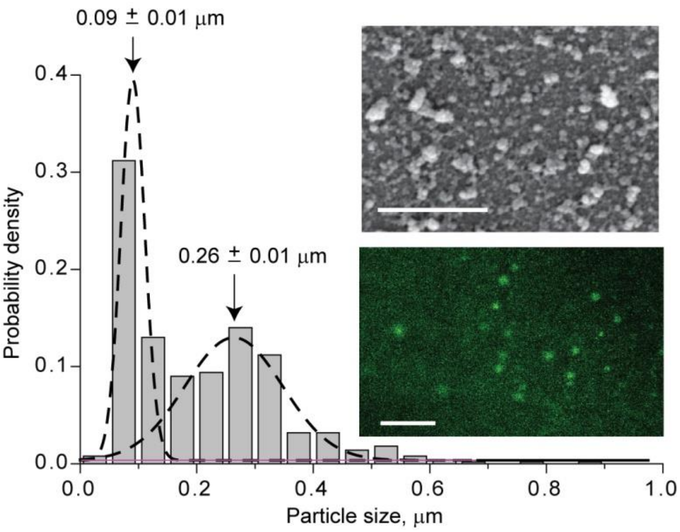

Figure S7. Characterisation of the filtered material. Size distribution of the particles retained on the surface of a 0.1-μm polycarbonate filter after filtration of PFP measured from the scanning electron micrographs (*upper inset*). *Lower inset*: Fluorescent confocal microscopy of the same filter stained for CD61 with FITC-labeled antibodies (magnification bars in the upper and lower insets = 2 μm). The size measurement of a total of 500 particles was performed in representative areas on three different filters.

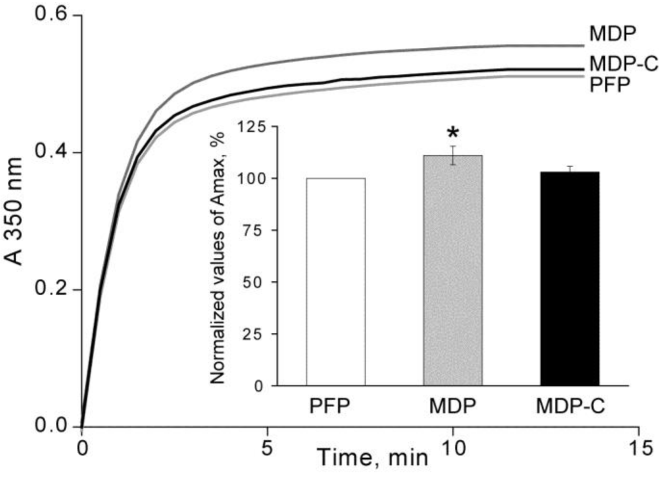

Figure S8. Effects of MPs on thrombin-induced fibrin formation. (A) Representative dynamic turbidity curves obtained in the cognate PFP, MDP and MDP-C plasma samples during fibrin formation induced by 0.1 U/ml thrombin in the absence of Ca^{2+}. (B) Values of the maximum clot turbidity relative to PFP. The results are presented as the mean value ± SD from the measurements performed in the plasma variants (PFP, MDP and MDP-C) obtained from the same blood samples (n=4).

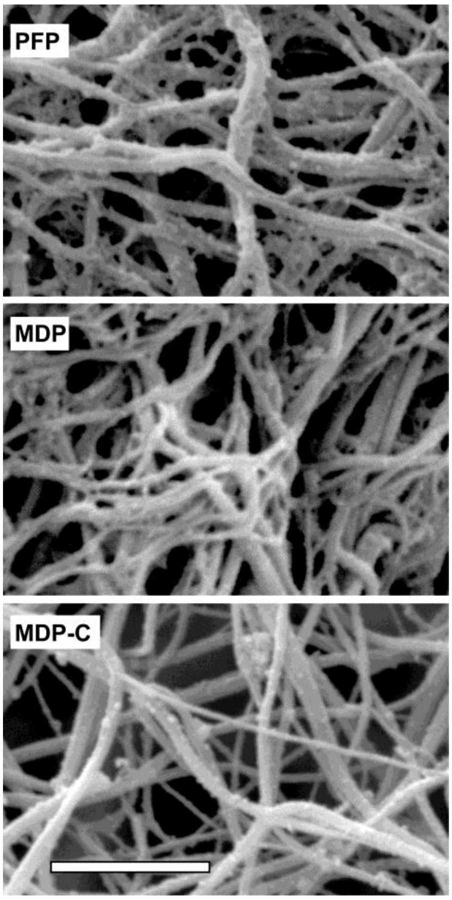

Figure S9. Fibrin clot ultrastructure upon thrombin-induced fibrin formation. Scanning electron micrographs of fibrin clots formed from the PFP, MDP and MDP-C samples, obtained upon thrombin-induced fibrin formation. Magnification bar = 2 µm.

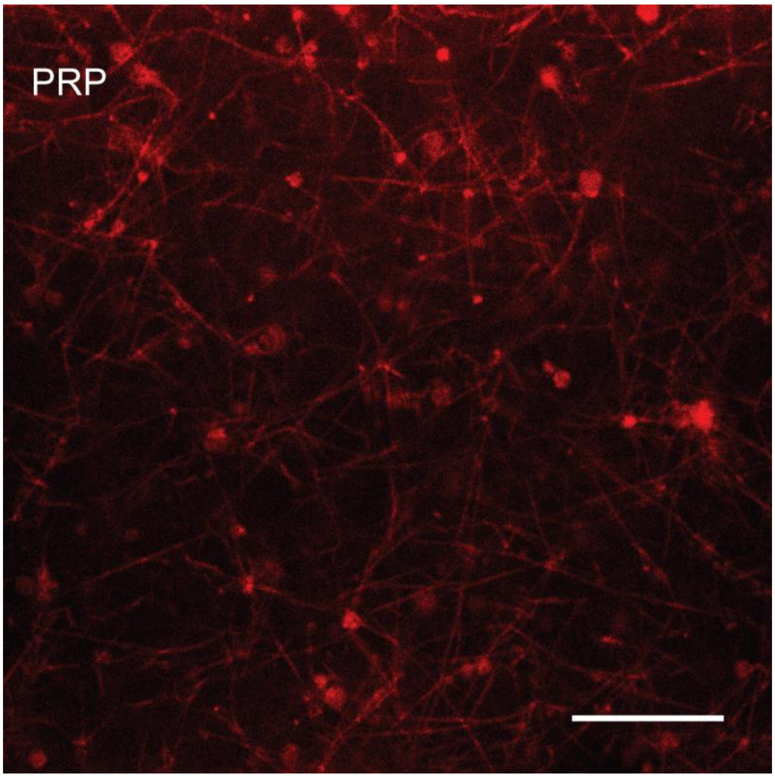

Figure S10. Visualization of platelets associated with fibrin fibres (positive control for the CD61 staining)**.** A fluorescent confocal microscopy image of a clot obtained from PRP and stained for CD61 with Alexa 647-labeled antibodies. Large fluorescent spots embedded into the polymeric network are revealed, demonstrating the presence of platelets associated with fibrin fibres. The smaller granularities on the fibrin fibres are likely MPs derived from the platelets. The magnification bar is 20 micrometers.

Supplementary Tables

Table SI. Effects of MPs on fibrin polymerisation and clot turbidity

Parameters (see Fig. S2)	*PFP*	*MDP*	*MDP-C*
Lag time (min)	7.8±3.4	28.8±12.1**	4.0±0.9
V (OD units/min)x10^{-3}	108±2	76±15*	134±12
A_{max} (OD units)	2.5± 0.2	2.8±0.1*	2.6±0.1

*p<0.05, **p<0.01 compared to PFP and MDP-C

Table SII. Effect of MPs on thrombin generation

Parameters (see Fig. S3)	*PFP*	*MDP*	*MDP-C*
Lag time (min)	10.0±3.3	26.7±6.0*	8.3±1.0
TTP (min)	24.4±3.9	55.1±16.0*	22.7±5.3
PTG (nM)	109.7±1.4	104.3±4.1	116.0±6.9
ETP (nM*min)	1271±45	1285±13	1306±59

*p<0.05 compared to PFP and MDP-C

Table SIII. Effect of MPs on internal fibrinolysis

Parameters (see Fig. S5)	*PFP*	*MDP*
Total lysis time (min)	809±36	650±51**
V (OD unit/min)x10^{-4}	78±11	104±8**
T ½ (min)	639±44	497±44**
CLT (min)	625±55	453±51**

**p<0.01 compared to PFP

Table SIV. Effect of MPs on external fibrinolysis

Parameters (see Fig. S6)	*PFP*	*MDP*
Lag time (min)	12.6±2.7	5.3±0,8*
Final degree of lysis (%)	48±11	77±8*
Average lysis rate (% lysis per 1 min)	0.5±0.1	0.8±0.1*

*p<0.05 compared to PFP
